# Influence of Maternal Factors (Weight, Body Condition, Parity, and Pregnancy Rank) on Plasma Metabolites of Dairy Ewes and Their Lambs

**DOI:** 10.3390/ani9040122

**Published:** 2019-03-28

**Authors:** Jose Luis Pesántez-Pacheco, Ana Heras-Molina, Laura Torres-Rovira, María Victoria Sanz-Fernández, Consolación García-Contreras, Marta Vázquez-Gómez, Pablo Feyjoo, Elisa Cáceres, Millán Frías-Mateo, Fernando Hernández, Paula Martínez-Ros, Juan Vicente González-Martin, Antonio González-Bulnes, Susana Astiz

**Affiliations:** 1 School of Veterinary Medicine and Zootechnics, Faculty of Agricultural Sciences, University of Cuenca, Avda. Doce de Octubre, 010220 Cuenca, Ecuador; jose.pesantez@ucuenca.edu.ec; 2 Department of Animal Reproduction, Instituto Nacional de Investigaciones Agrarias y Alimentarias (INIA), Avda Pta. de Hierro s/n, 28040 Madrid, Spain; andelash@ucm.es (A.H.-M.); torrerovi@gmail.com (L.T.-R.); mvsanzfernandez@gmail.com (M.V.S.-F.); garcia.consolacion@inia.es (C.G.-C.); bulnes@inia.es (A.G.-B.); 3 Technical Department, Granja Cerromonte SL, 05358 San Juan de la Encinilla, Ávila, 05358, Spain; granja@cerromonte.es; 4 Faculty of Veterinary Medicine, Complutense University of Madrid (UCM), Avda. Pta. de Hierro s/n, 28040 Madrid, Spain; mvgomez@ucm.es (M.V.-G.); feyjoo96@gmail.com (P.F.); elicacer@ucm.es (E.C.); millanfr@ucm.es (M.F.-M.); juanvi@vet.ucm.es (J.V.G.-M.); 5 Departamento Producción y Sanidad Animal, Salud Pública Veterinaria y Ciencia y Tecnología de los Alimentos (PASAPTA), Facultad de Veterinaria, Universidad Cardenal Herrera-CEU, Tirant lo Blanc, 7. 46115 Alfara del Patriarca Valencia, Spain; paula.martinez@uchceu.es; 6 Technical Department, TRIALVET SL, C/ Encina 22, Cabanillas de la Sierra, 28721 Madrid, Spain

**Keywords:** high milk yield, dairy sheep, pregnancy, age, metabolomics, body condition score, birth weight

## Abstract

**Simple Summary:**

The present study assessed the effects of maternal parity, weight, body condition score (BCS), and pregnancy rank (single vs. multiple) on maternal metabolism during pregnancy and subsequent lactation, as well as on lamb birth weight, perinatal viability, and metabolism. The results highlight the relevance of appropriate nutritional management to maintain maternal BCS and offspring metabolism within physiological ranges, allowing sheep to face the metabolic challenges of lactation and pregnancy. Adequate nutrition and management reduce the influence of maternal factors on offspring phenotype.

**Abstract:**

Pregnancy and lactation are challenging states that affect maternal and lamb health. In Lacaune dairy sheep, we evaluated the impact of parity, pregnancy rank, and body condition on body weight and the condition of ewes and lambs in mid-pregnancy (75 ± 5 d), in late pregnancy (142 ± 4d), and postpartum (52 ± 5d pp). Maternal age was associated with initial decreases, followed by increases, in body weight and condition. After lambing, both mature and maiden ewes lost weight and body condition. Maternal indices of glucose, protein, and lipid metabolism were within physiological values during pregnancy, but postpartum values depended on maternal parity and pregnancy rank, with multiple-pregnant ewes showing a postpartum increase in glucose and maiden sheep a postpartum increase in plasma cholesterol concentration. Male lambs were heavier than female lambs at birth, and lambs born to mothers with higher body condition scores were heavier. Lambs born as singletons were heavier than those born in litters. Maternal age and pregnancy rank did not influence lamb metabolic indicators. Sex affected plasma concentrations of glucose, triglycerides, and cholesterol. Maternal metabolic indicators showed minimal effects on lamb phenotype. These results suggest that, when appropriately fed, dairy sheep can cover the metabolic demands of pregnancy and milk production, regardless of age and pregnancy rank.

## 1. Introduction

The productive performance of farms is highly dependent on adequate metabolic status of the animals. In dairy females, cows or sheep, the two stages of high metabolic demand (lactation and gestation) tend to be concurrent, since pregnancy is induced when animals are still producing milk [1]. During lactation, concomitant with early and mid-pregnancy, around 80% of the metabolites (i.e., free amino acids, glucose, and fatty acids) circulating in the blood are used for milk production [2]. During later stages of pregnancy, maternal metabolism focuses mostly on providing energy for fetal development and growth [3]. These increased metabolic challenges cannot be met through the intake of increased dry matter in the case of dairy cows or ewes, particularly those in late pregnancy and the early postpartum period. Instead, these animals adapt their metabolism of carbohydrates, proteins, and lipids [4,5] to maintain their own homeostasis and to channel glucose and other nutrients towards the pregnant uterus and the lactating mammary gland. These events have been intensively studied in cows, but there is a scarcity of data on dairy sheep. 

Body weight (BW) and body condition score (BCS) are often used as indicators of the appropriateness of a ewe’s nutrition and the level of energy reserves during mid-pregnancy and late pregnancy; these variables correlate with changes in circulating factors that manage the body’s energy reserves [6]. However, BW during late pregnancy is affected by the number of fetuses, and its usefulness as an indicator has other limitations [7,8]. More detailed knowledge of the metabolic status of ewes during the demanding periods of pregnancy and lactation may help identify more accurate ways to assess ewe nutrition and thereby optimize performance [9].

In dairy ruminants, the combination of high nutrient demand and low intake during early lactation and late pregnancy can give rise to negative energy balance, which substantially increases the risk of metabolic diseases [10], particularly ketosis. Pregnant and lactating dairy ewes frequently develop ketosis, as can ewes in late pregnancy (which are no longer lactating); this ketosis contributes to significant production losses. In sheep, lipid mobilization may begin several weeks before lambing, especially in ewes with multiple pregnancies [11]. Catabolism of amino acids increases in mothers, involving protein breakdown in skeletal muscles [2,9,12] at the expense of protein synthesis [4], as well as recycling urea into the rumen and decreasing its urinary excretion [13]. This breakdown makes amino acids available, which are used as a gluconeogenic or ketogenic substrate in the liver [14]. 

Inadequacy of energy balance in pregnant sheep is dangerous not only for the mothers but also for the pre- and perinatal viability and performance of lambs [15,16]. Restricted metabolite availability during pregnancy can increase risk of low birth weight and perinatal mortality [4,12]. 

Fetal development is influenced by genetic and maternal factors, which can interact with one another. Maternal physiology and placental function exert a profound effect on fetal growth and account for over 30% of the variation in birth weight [17]. Maternal nutrition is essential for an adequate supply of nutrients and environmental products required for fetal development [18]. Maternal under- and overnutrition can significantly affect lamb prenatal growth, development, and productive performance through prenatal programming [19,20]. In addition to inadequate maternal nutrition, other maternal factors (BW, age, parity) can influence lambs' ability to fulfil their metabolic requirements [21,22]. Indeed, mother and fetus compete for the nutrients needed for maternal body development (primiparous ewes) or for lactation (multiparous ewes). Pregnancy rank (single vs. multiple) can exacerbate the negative effects of maternal factors, reflecting the limited ability of the mother to supply nutrients to the fetus in multiple pregnancies [21]. 

We hypothesized that the effects of maternal factors (i.e., parity, weight, body condition, and pregnancy rank) on maternal metabolism and characteristics of the offspring may be exacerbated in high-yielding dairy ewes. The available information is, however, very scarce. Therefore, the present study analyzed a single, adequately fed, high-producing, Lacaune dairy sheep flock to explore the influence of maternal age or parity (maiden vs. mature ewe), pregnancy rank (single vs. multiple), and maternal body condition score (thin vs. average vs. fat) on maternal metabolism in mid-pregnancy, in late pregnancy, or postpartum as well as on offspring metabolism and BW. This comprehensiveness will help us to better assess the health and productivity of high-yielding dairy sheep.

## 2. Materials and Methods

### 2.1. Animals and Handling 

**Figure 1:**
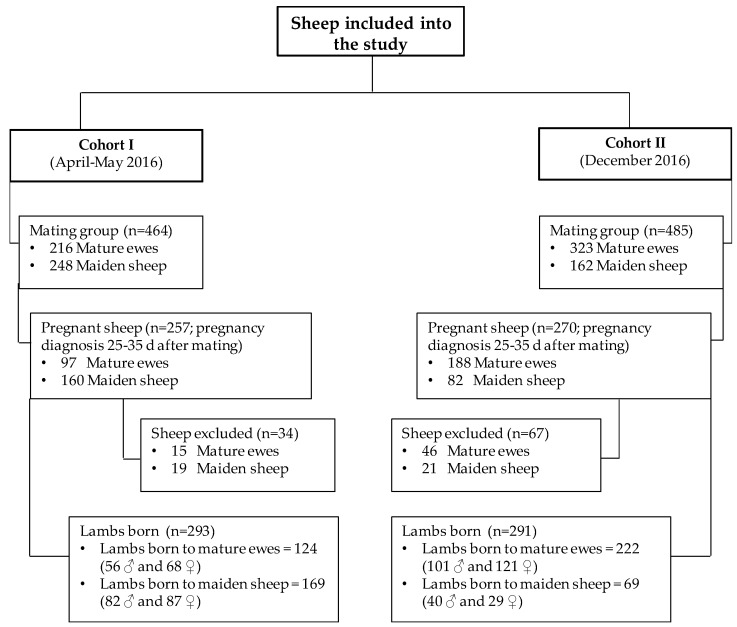
Scheme illustrating sheep inclusion in the different cohorts to follow sheep pregnancy, lambing, the postpartum period, and the neonatal life of the studied individuals at a commercial, high-yielding Lacaune dairy farm.

The study was carried out on a flock of high-yielding Lacaune dairy sheep located at a single commercial farm (Cerromonte Farm, San Juan de la Encinilla, Avila, Spain; continental weather, latitude of 40.90 N, altitude of 900 m above sea level). Sheep were housed indoors with outdoor access, so they were exposed to seasonal changes in photoperiod and temperature. Production parameters are summarized in Table 1, while criteria for selection of animals are indicated in Figure 1. According to their production level, they received unifeed mixtures (total mixed ration system) containing corn, soybean, dried beet pulp, alfalfa, rye-silage, and wet brewer’s grain. Sheep were monitored for adequate health status and specific pathogens and were milked twice a day. 

Pregnancies in mature and maiden sheep occurred after 14-day treatment with intravaginal progestogen-impregnated sponges (20 mg fluorogestone acetate, FGA; Chronogest^®^, MSD AH, Boxmeer, Netherlands). On the day of sponge removal, ewes received 400 i.u. of eCG (Foligon^®^, MSD AH) and immediately afterwards were exposed to natural mating with proven rams, 2–5 years old, at a rate of 1:5 for 28 days. Ram fertility was checked individually by routine testing of ram reproductive capacity (libido, mounting capacity, sperm motility, and concentration), as performed by the farm veterinarian. 

Sheep lambed in collective pens, and the lambs were weaned on the day after birth and then fed with formula (ELVOR 62, Sofivo, Saint Brice en Coglès, France) in a closed “weaning unit” for 18–25 days. The weaning unit had a capacity of 960 newborn lambs, divided into pens containing 60 lambs each. Each pen was equipped with slatted flooring, temperature control (minimum, 17 °C; maximum, 27 °C), separate ventilation systems, and automatic lamb feeders. 

### 2.2. Study Design and Measured Variables

This study was prospective, randomized, observational, and cohort-based (Figure 1). Two mating groups (both including mature and maiden sheep) were monitored during two reproductive seasons (April–May and December). Adult sheep were between 2 and 5 years old, with an average of 3.5 years old, and were evenly distributed among groups. Pregnancy was diagnosed by transabdominal ultrasonography (NanoMax, Sonosite, Bothell, WA, USA) at 25–35 days after ram introduction in order to select cohorts of pregnant sheep with similar lambing dates.

Altogether the study included 527 pregnant ewes (285 mature and 242 maiden), from which 34 were lost from the first mating group and 67 lost from the second group during late pregnancy or parturition because of death (8 ewes), abortion (3), non-pregnancy (7), lambings after the date predicted (3), or an absence of identification of lambs born (80). Subsequently, a total of 584 lambs (305 females and 279 males) were included in the study, of which 346 were born to mature ewes and 238 to maiden ewes. A global lamb mortality of 5.82% was observed (34/584 lambs), and 550 lambs could be measured and sampled at the age of 17 days.

#### Parameters recorded and used to categorize and compare sheep and lambs

The parameters recorded and used to study the population were as follows.

- Pregnancy rank: single vs. multiple (diagnosed by ultrasonography and verified after lambing)
- Parity: maiden sheep vs. adult sheep
- Body condition score (BCS), as assessed by two trained observers using the 5-point scale (1 = emaciated, 5 = obese) [23]. BCS was determined three times: in mid-pregnancy (BCS-1; 75 ± 5 days of pregnancy), in late pregnancy (BCS-2; 142 ± 4 days of pregnancy), and postpartum (BCS-3; 52 ± 5 days after lambing). On the basis of BCS, sheep were divided into three groups: thin (BCS ≤ 2), average (2 < BCS < 3), or fat (BCS ≥ 3).
- Lamb parameters:
  ○ Sex of the lamb: Male vs. female
  ○ Sex of sibling(s) (in multiple lambings)
    ▪ Male and female siblings
    ▪ Only male sibling(s) (same as the lamb)
    ▪ Only female sibling(s) (same as the lamb)
  ○ Lamb birth weight
    - Normal-birth-weight lambs: Lambs with birth weight >3 kg
    - Low-birth-weight lambs: Lambs with birth weight ≤3 kg. This was equivalent to one standard deviation below the mean birth weight of all lambs [24,25].

### 2.3. Metabolic Parameters of Sheep

**Figure 2:**
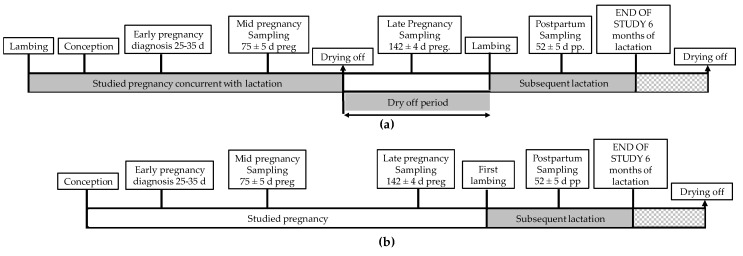
Timeline of monitoring of (**a**) pregnant adult sheep and (**b**) pregnant maiden sheep. Measurements were taken three times (in mid-pregnancy, in late pregnancy, and postpartum) distributed over the “studied” lactation and pregnancy as well as the “subsequent” lactation.

All ewes were sampled for metabolic testing at the same three times when BCS was determined (Figure 2). BW was individually recorded using an electronic balance (TRU-TEST, Auckland, New Zealand). Fasting plasma samples were collected via jugular venopuncture into standard 10 mL ethylene diamine tetra-acetic acid (EDTA) vacuum tubes (Vacutainer^®^ System Europe; Becton Dickinson, Meylan, France). Blood samples were centrifuged at 1500 *g* for 15 min, and the plasma was stored in polypropylene vials at −80 °C for later metabolic assays using a clinical chemistry analyzer (Saturno 300 plus, Crony Instruments s.r.l., Rome, Italy) according to the manufacturer's instructions. The following biochemical parameters were measured as metabolic indicators: plasma glucose (GLU), lactate (LAC), cholesterol (CHO), triglycerides (TGL), β-hydroxybutyrate (β-OHB), non-esterified fatty acids (NEFA), and urea (UR). 

### 2.4. Assessment of Early Postnatal Development and Metabolic Features of Lambs

Immediately after lambing, 584 living lambs were labelled with ear tags indicating the mother, and they were sexed and weighed. A total of 88 adult sheep had singletons while 134 had twins, and 104 maiden sheep had singletons while 86 had twins. At the age of 17 ± 5 days, a total of 34 lambs had died (5.82% total mortality rate); 16 dead lambs were born to mature ewes (16/346; 4.62% mortality rate in mature ewes) and 18 to maiden sheep (18/238; 7.56% mortality rate in maiden sheep). A total of 19 dead lambs were male (19/279; 6.8%, and 15 were female (15/305; 4.92%). At this second time point, lambs were weighed, and body-length was measured using a standard measuring tape. Trunk length was defined as the distance of the spine from the withers to the base of the tail. Body mass index (BMI) was calculated [birth weight in (kg)/length in (m)^2^] as described [26]. Thoracic and abdominal circumferences were measured only at the second time point (17 ± 5 days old). At this second time point, blood samples were taken via jugular venopuncture into standard 5 mL EDTA vacutainer tubes and stored at −80 °C until a later assay of the same metabolic parameters as in adult and maiden sheep. 

### 2.5. Statistical Analyses 

Data were analyzed using SPSS^®^ 22.0 (IBM, Armonk, New York, USA) by the Statistical Department of the Center for Research Support of Complutense University of Madrid, Spain. Changes over time in BW, BCS, BMI, length, and metabolic indices of sheep and lambs were assessed for significance using Analysis of Variance (ANOVA) for repeated measures in a general linearized model with Greenhouse–Geisser correction. Differences between groups at different time points were assessed for significance using non-parametric analysis, the Kruskal–Wallis test or the Mann–Whitney test, after confirming the skewed (non-normal) distribution of the data. Inter-group differences in body circumference, average daily gain, and metabolic indices in lambs were also assessed for significance using the independent *t* test. Pearson correlation coefficients were calculated to assess the strength of potential relationships between sheep variables, or of relationships of lamb birth weight with maternal metabolic indices. Data were expressed as mean ± S.E.M., and differences were considered significant if *p* < 0.05. Differences associated with *p* between 0.05 and 0.09 were defined as tendencies. 

## 3. Results

### 3.1. Changes in Maternal BW and BCS Throughout Pregnancy

**Figure 3:**
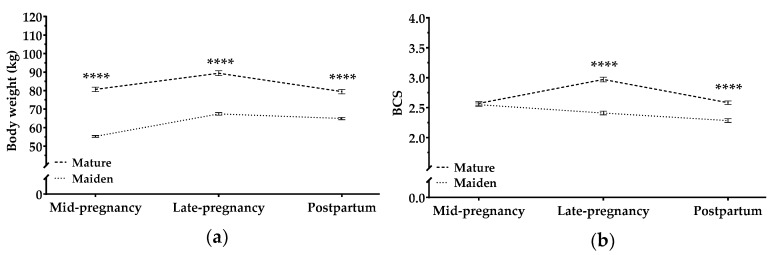
Body weight (kg) (**a**) and body condition score (BCS) (**b**) in mature ewes (dashed line) and in maiden sheep (dotted lines) throughout the study period. Data are mean ± S.E.M. Time × age-group interaction was significant (*p* < 0.0001). Asterisks indicate significant differences between age groups at each time point (**** *p <* 0.0001).

BW and BCS were higher in mature ewes than in maiden ewes at all time points (*p* < 0.0001; Figure 3). Both groups increased significantly in BW during pregnancy (both *p* < 0.0001), but only mature sheep increased significantly in BCS (*p* < 0.0001). 

Maternal BW was higher and increased more steeply in late stages of multiple pregnancies than in late stages of single pregnancies (*p* < 0.001; Figure S1). Pregnancy rank did not, however, significantly affect maternal BCS.

### 3.2. Changes in Maternal Metabolic Indices Throughout Pregnancy 

#### 3.2.1. Glucose

**Figure 4:**
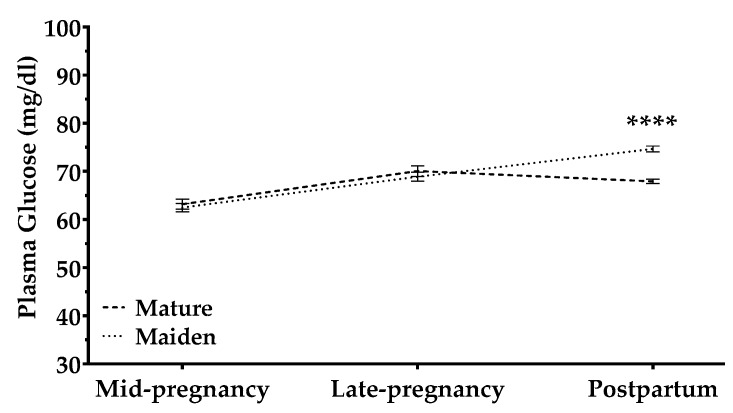
Plasma glucose concentrations (mg/dL) in mature ewes (dashed line) and maiden ewes (dotted lines) throughout the study period. Data are mean ± S.E.M. The time × age-group interaction was significant (*p* < 0.0001). Asterisks indicate significant differences between age groups (**** *p <* 0.0001).

Maternal plasma glucose increased during pregnancy (Figure 4): levels were higher and the increase steeper in maiden than in mature sheep at late pregnancy (significant interaction time × age). This increase was modulated by pregnancy rank, independently of age: compared to multiple pregnancies, single pregnancies showed higher glucose concentration throughout gestation and a sharper increase in late gestation.

Maternal age and BCS interacted to affect plasma glucose concentrations throughout pregnancy. Maiden sheep, but not mature sheep, with high BCS in mid-pregnancy showed higher plasma glucose concentrations during mid and late pregnancy than did those with lower and average BCS. In contrast, mature ewes, but not maiden sheep, with high BCS in late pregnancy showed higher plasma glucose concentrations at late pregnancy than did ewes with lower BCS (Figure S2). 

#### 3.2.2. Lactate

**Figure 5:**
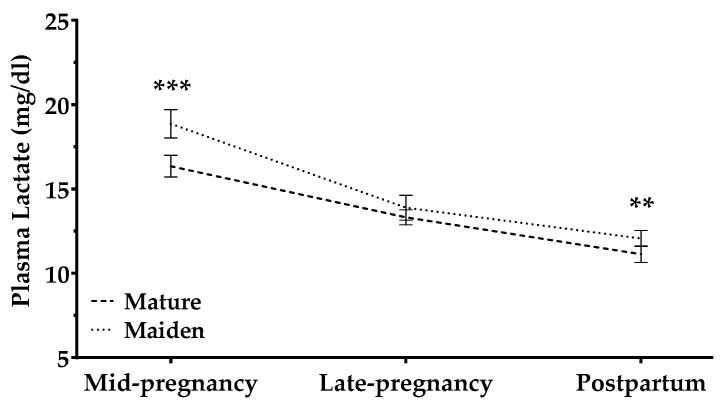
Plasma lactate concentrations (mg/dL) in mature sheep (dashed line) and maiden sheep (dotted lines) throughout the study period. Data are mean ± S.E.M. Asterisks indicate significant differences between age groups (*** *p* < 0.001; ** *p* < 0.01).

Maternal plasma lactate concentrations decreased during and after pregnancy in all the animals, independently of age (Figure 5). However, lactate levels were affected by pregnancy rank: the decrease in levels during pregnancy tended to be steeper for single pregnancies than for multiple ones (*p* = 0.07). 

Plasma lactate concentrations were affected by BCS in late pregnancy (BCS-2) in both mature and maiden sheep: a lower BCS-2 was related to lower lactate levels in late pregnancy and to higher levels postpartum (*p* < 0.05). Lactate concentrations were affected by BCS-3 only in maiden sheep: fatter females showed a significant increase in lactate at late pregnancy (*p* < 0.05; Figure S3).

#### 3.2.3. Cholesterol and Triglycerides

**Figure 6:**
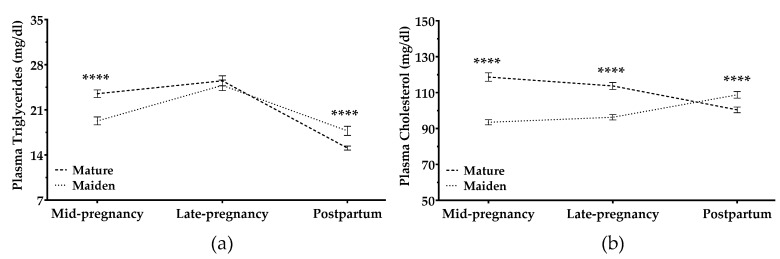
Plasma concentrations (mg/dL) of (**a**) triglycerides and (**b**) cholesterol in mature ewes (dashed line) and maiden ewes (dotted lines). The time × age-group interaction was significant (*p* < 0.0001). Asterisks indicate significant differences between age groups at each time point (**** *p* < 0.0001).

Maternal plasma concentrations of triglycerides and cholesterol increased during pregnancy (*p* < 0.0001; Figure 6a,b). Both parameters were higher in maiden than mature ewes during the entire pregnancy. Triglycerides decreased in all sheep after lambing, and this decrease was greater in mature ewes (Figure 6a). In contrast, cholesterol decreased in mature but not maiden ewes (Figure 6b).

Pregnancy rank did not significantly affect lipid metabolism during pregnancy in either mature or maiden sheep. After pregnancy, rank did not affect lipid metabolism in mature sheep. In maiden sheep, however, the concentrations of cholesterol and triglycerides were greater in multiple pregnancies than in single pregnancies (*p* = 0.03 and *p* = 0.009, respectively).

Plasma cholesterol concentrations were significantly influenced by BCS at mid-pregnancy (BCS-1) in mature sheep (*p* = 0.02) and maiden sheep (*p* < 0.001; Figure S4). However, the effects of BCS at late pregnancy (BCS-2) were found only in mature ewes: lower BCS-2 was related to lower cholesterol concentrations. Postpartum BCS showed an effect only in maiden sheep: fat maiden sheep showed significantly lower cholesterol levels (*p* > 0.0001). Triglycerides decreased after pregnancy in all animals; this decrease was steeper in maiden sheep with single pregnancies than in maiden sheep with multiple pregnancies or in mature ewes of either pregnancy rank (Figure S4).

Plasma concentrations of triglycerides were affected by BCS-2, independently of age: ewes with higher BCS-2 showed higher plasma concentrations during pregnancy (*p* < 0.001). After pregnancy, plasma triglycerides declined in all ewes; ewes with higher BCS-2 and BCS-3 were associated with a steeper decrease in plasma triglycerides postpartum than were thinner ewes (Figure S5).

#### 3.2.4. β-Hydroxybutyrate and Non-Esterified Fatty Acids

**Figure 7:**
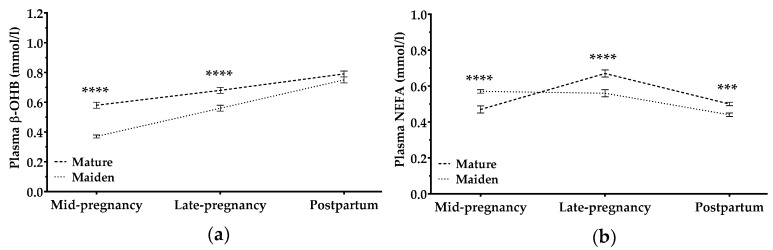
Concentration of **(a)** β-hydroxybutyrate (β-OHB) and (**b**) non-esterified fatty acids (NEFA) in plasma (both mmol/L) in mature ewes (dashed line) and maiden sheep (dotted lines) throughout the study period. The time × age-group interaction was significant (*p* < 0.0001). Data are mean ± S.E.M. Asterisks indicate significant differences between age groups at each time point (**** *p* < 0.0001; *** *p* < 0.001).

Concentrations of β-hydroxybutyrate (β-OHB) and non-esterified fatty acids (NEFA) in plasma changed in different ways during pregnancy. Plasma β-OHB levels increased during and after pregnancy in mature and maiden sheep; the increase was significantly steeper in maiden sheep (*p* < 0.0001; Figure 7a). In mature ewes, NEFA levels increased during pregnancy and decreased postpartum; in maiden sheep, NEFA levels decreased steadily with pregnancy time and decreased further after pregnancy (*p* < 0.001; Figure 7b).

Pregnancy rank and BCS had no significant effects on changes in β-OHB, with the following exceptions: (1) mature ewes with multiple pregnancies showed higher values of β-OHB at late pregnancy and postpartum than those with single pregnancies (*p* < 0.05; Figure S6); (2) ewes with lower BCS-2 and BCS-3 showed a peak in β-OHB concentration at the end of gestation (*p* < 0.0001; Figure S6). Maiden sheep were not affected by different BCS levels: β-OHB concentrations increased during and after pregnancy in all these animals (Figure S6).

Plasma NEFA concentrations in mature ewes were affected by pregnancy rank: mature ewes with multiple pregnancies showed a steeper increase in late pregnancy than did those with single pregnancies (Figure S7). In contrast, BCS did not significantly affect NEFA levels at any time point with one exception: mature ewes with low BCS-2 showed higher levels of NEFA at mid-gestation than did those ewes with higher BCS-2 (Figure S7).

#### 3.2.5. Urea

**Figure 8:**
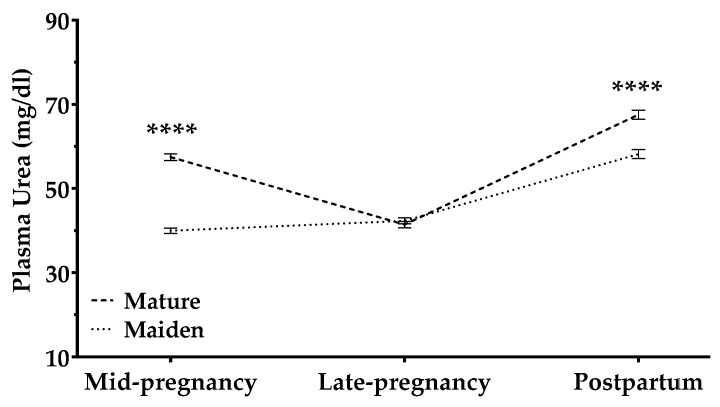
Plasma urea concentrations (mg/dL) in mature ewes (dashed line) and maiden sheep (dotted lines) throughout the study period. Data are mean ± S.E.M. The time × age-group interaction was significant (*p* < 0.0001). Asterisks indicate significant differences between age groups at each time point (**** *p* < 0.0001).

Plasma urea levels were affected by a significant interaction between sheep age and pregnancy stage (*p* < 0.0001). In mature ewes, urea levels decreased throughout pregnancy and increased after pregnancy; maiden sheep showed a slight, constant increase during pregnancy and postpartum (Figure 8). This increase was steeper after lambing than between mid and late pregnancy.

Pregnancy rank affected plasma urea only in maiden sheep: ewes with single pregnancies showed a slight increase in urea levels during pregnancy, which then increased postpartum more sharply than those in maiden sheep with multiple pregnancies (*p* < 0.05; Figure S8). In a similar way, BCS affected urea depending on an interaction between sheep age and pregnancy stage: significant effects were observed in maiden sheep at mid-pregnancy (BCS-1; *p* < 0.001) and in mature ewes in late pregnancy (BCS-2; *p* < 0.05). In contrast, postpartum BCS (BCS-3) affected mature and maiden sheep urea levels (*p* < 0.05; Figure S8). Postpartum urea concentrations increased more steeply in maiden sheep that were fatter at mid-pregnancy (BCS-1) and postpartum (BCS-3); postpartum urea concentrations in mature ewes fatter in late pregnancy (BCS-2) increased more steeply than those in thinner ewes (Figure S8).

### 3.3. BW, Body Size, and Metabolic Phenotype of Lambs

Lamb body weight and size depended on sex (Table 1): male lambs were heavier and larger than females at birth and 17 days old. Sex also affected metabolism: male lambs showed higher plasma concentrations of glucose and triglycerides and lower concentrations of cholesterol than did female lambs (*p* < 0.05; Table 2).

Birth weight affected the morphological and metabolic phenotype of lambs: low-birth-weight lambs showed lower NEFA and urea concentrations but higher cholesterol concentration in plasma than normal-birth-weight lambs (Table 3).

Maternal features also affected lamb birth weight and postnatal development (Table 4 and Tables S1 and S2): mature ewes gave birth to heavier lambs than did maiden ewes, and ewes with single pregnancies gave birth to heavier lambs than did ewes with multiple pregnancies. Lamb BW was affected by maternal age and pregnancy rank: significant interactions were observed for time × age group (*p* < 0.0001) and for time × pregnancy rank (*p* = 0.04; Table 3). By 17 days, BW was similar between lambs born as single or multiple pregnancies. Nevertheless, lambs born to sheep with multiple pregnancies showed only marginally greater average daily weight gain than did sheep with single pregnancies (*p* = 0.62; Table 3).

Birth weight and birth trunk length were not affected by BCS at mid-pregnancy (BCS-1), but they were influenced by BCS in late pregnancy (BCS-2; *p* < 0.0001; Table S2). Lambs born to sheep with high BCS-1 and BCS-2 were heavier and larger than those born to sheep with medium or low BCS-1 and BCS-2 (*p* < 0.001 for BCS-1 and *p* < 0.0001 for BCS-2). By 17 days, BCS-1 did not show a significant influence, but BCS-2 did (*p* < 0.0001; Table S2).

Pregnancy rank did not significantly influence metabolic features of lambs. Similarly, maternal age did not significantly influence plasma concentrations of glucose, cholesterol, β-OHB, or NEFA in lambs, except that lambs born to mature ewes showed lower triglycerides and lactate and higher urea concentrations (all *p* < 0.005; Table 3). Lambs born to ewes with high BCS-1 and BCS-2 showed higher plasma β-OHB concentrations at 17 days than lambs born to ewes with medium or low BCS-1 (*p* < 0.005) and BCS-2 (*p* < 0.05; Table S2). 

Possible correlations were explored among maternal and fetal metabolic features (Tables S3 and S4). Lamb birth weight positively correlated with maternal β-OHB (*r* = 0.212 *p* < 0.0001) and urea (*r* = 0.248 *p* < 0.0001) at mid-pregnancy, independently of maternal age and BCS. Among maiden sheep in late pregnancy, only triglyceride concentrations showed a positive, significant correlation with birth weight (*r* = 0.289 *p* < 0.0001).

## 4. Discussion

The present study suggests that the magnitude of the metabolic challenges imposed by pregnancy depends on maternal age and pregnancy rank in dairy ewes. Similar results have been reported in meat ewes [3,6,11]. Maternal BW increased in maiden and adult sheep during pregnancy, but maiden sheep lost BCS during pregnancy and after lambing, suggesting a loss of conceptus-free live weight. These results are consistent with previous work on pregnant maiden sheep [27,28]. On the study farm, the first mating was induced between 8 and 10 months of age, so mean age at first lambing was 14.4 months [29], which is an age when maiden sheep have to finalize their growth. These maiden sheep face the challenge of finishing their own growth [22], while also fulfilling fetal needs. Mature dairy ewes, in contrast, can cover fetal nutrient demand better than maiden sheep in late pregnancy, since they are already dried-off. After lambing, both maiden and mature sheep lost BW and BCS as a consequence of milk production, which reflects the high-yielding dairy condition of these animals in the study farm. These results support the idea that high-yielding dairy ewes begin lactation with a reduced intake capacity that leads to negative energy balance, as documented extensively in dairy ruminants [11,30]. 

Consistent with a greater metabolic challenge for maiden than for mature ewes, we found that fasting glucose in younger animals increased postpartum in tight association with BCS: only “fatter” maiden sheep with high BCS during pregnancy and after lambing showed increases in plasma glucose [31,32,33]. This may mean that fatter maiden animals did not regulate glucose levels as well as thinner maiden sheep. Nevertheless, glucose levels in all animals in our study were within the physiological range for sheep (32.42–79.99 mg/dl) [34,35,36,37]. This suggests that nutritional management on the study farm was appropriate.

Pregnancy rank did not influence maternal BW or BCS. Similarly, other studies failed to find a clear effect of multiple pregnancies on such parameters or on glucose levels during pregnancy and in the early postpartum period [38,39]. Our results may reflect that, under productive conditions, the Lacaune breed does not show high prolificacy, with an average of 1.6 lambs/lambing [40,41]. They may also reflect that nutrition management on the study farm fully satisfied the metabolic needs of the ewes. 

Pregnancy rank did appear to affect the ability of our ewes to maintain stable glucose levels. The increase in glucose from pregnancy to the postpartum period was greater in sheep with multiple pregnancies than in those with single pregnancies, regardless of whether the sheep were maiden or mature. This greater availability of glucose may reflect an adaptation to the presence of multiple fetuses in the high-producing Lacaune dairy breed [41]. It may also result from the greater release of cortisol after multiple lambings previously observed in meat crossbred ewes [42]. Studies in Würtenberg breeds have also reported higher levels of plasma glucose in milk-producing ewes than in non-lactating, pregnant ewes [43]. Whatever the cause, the greater glucose availability with multiple pregnancies likely helps ensure that the metabolic needs of all fetuses are met: risk of hypoglycemic stress is higher in ewes bearing twins than in ewes bearing single lambs [44]. 

The sheep in our study maintained constant concentrations of cholesterol and triglycerides during pregnancy. After lambing, both mature and maiden sheep showed a decrease in triglycerides. Cholesterol levels after lambing depended on maternal age: they decreased in mature ewes but increased in maiden sheep, with a steeper increase in maiden sheep with multiple pregnancies than in maiden sheep with single pregnancies. However, studies in other meat breeds have reported no significant changes in plasma lipid metabolites of ewes during pregnancy, and even a decrease in late pregnancy and after lambing [43,45]. Triglycerides in the blood are fuel sources [19,46] that are likely consumed when energy requirements increase, such as in late pregnancy. This would explain the postpartum decrease. Our results suggest that high-yielding maiden dairy sheep rely less on lipid resources for energy than do mature sheep.

Maternal lipid metabolism was affected by their BCS: sheep with higher BCS during pregnancy and postpartum showed greater plasma concentrations of cholesterol and triglycerides than did sheep with lower BCS, although these values were within physiological levels for all animals, cholesterol (from 65.01 ± 6.78 to 89.01 ± 7.90 mg/dL) and triglycerides (17.7–25.66 mg/dL) [37,47]. Consistently, a study in dairy cows found that adequate BCS at the end of pregnancy was associated with higher levels of metabolites [48], and another study found similar results in non-pregnant ewes [13]. At least one study has reported no significant variation in glucose or cholesterol levels of ewes with different BCS [49], but those results are difficult to compare with ours, since they are based on samples taken from ewes after mating as well as in late pregnancy. When interpreting our results and comparing them with those from other studies, it is important to keep in mind that most of our sheep had appropriate BCS throughout the observation period, with a mean BCS-1 of 2.56 ± 0.44, a mean BCS-2 of 2.69 ± 0.52, and a mean BCS-3 = 2.45 ± 0.46, which likely explains why plasma lipid concentrations were always within physiological levels [37,47]. 

In contrast to lipid parameters, plasma lactate concentrations decreased with pregnancy stage and after pregnancy in all animals, independently of maternal age. The decrease was steeper in ewes with single pregnancies than in those with multiple pregnancies. Our results with lactate and glucose levels further support the idea that nutrition management in our cohort was appropriate, since adequately fed ewes show a precarious carbohydrate metabolism during late pregnancy and early lactation [50]. Deficiencies in carbohydrate metabolism was more severe among the thinnest ewes (based on BCS in late pregnancy); BCS-2 interacted significantly with lactate concentration × time. 

Animals may catabolize proteins in order to cover nutrient demand, which translates to increases in urea levels in plasma and milk [51]. Interestingly, the increase in plasma urea concentrations occurred after lambing and not in late pregnancy. This is similar to results observed in meat ewes [38] and to a report of higher serum concentrations of urea and total protein during lactation than in late pregnancy in ewes [43]. BCS appears to influence protein metabolism after lambing: maiden and mature sheep with higher BCS showed a steeper plasma urea increase postpartum than did thinner animals. Similar results were observed in dairy cows: higher BCS at calving was associated with higher urea levels postpartum [52,53,54].

Overall, our results indicate good adaptation by maiden and mature ewes to cover nutrient demands during pregnancy, even in multiple pregnancies. Our study also examined how ewes responded to the metabolic demands posed by lactation. Early lactation creates negative energy balance [55,56], which we confirmed in our sheep by assessing concentrations of β-OHB and NEFAs. β-OHB concentration increased with pregnancy time and after lambing in maiden and mature ewes, although younger animals showed a steeper increase. In contrast, pregnancy rank did not affect β-OHB concentrations over time. A study in meat ewes also reported an increase in β-OHB concentrations during pregnancy [38]. On the other hand, their results differed from ours in that they detected higher β-OHB levels in twin-bearing ewes than in single-bearing animals, and they found that β-OHB levels decreased one month after lambing. These differences may reflect that the ewes in that study were not dairy sheep; our high-producing dairy sheep were likely under greater metabolic challenge than those sheep. 

We observed that only mature ewes with a low BCS in late pregnancy showed a peak in β-OHB levels in late pregnancy and a steep decrease after lambing. This may mean that these ewes are more challenged during the last phase of fetal growth than during lactation. This may be also due to the fact that these ewes gave slightly lower levels of milk after lambing (1.41 ± 0.53 L/d in thin ewes vs. 2.28 ± 0.73 L/d in fat ewes), in order to decrease their global metabolic challenge and to be able to cover fetal demands adequately.

In our study, NEFA concentrations in maiden sheep decreased over time, while the levels in mature sheep increased during pregnancy and then decreased after lambing. NEFAs reflect the level of lipid catabolism, and their concentration increases when glucose metabolism is deficient. After lambing, a simultaneous decrease in NEFA levels and an increase in β-OHB levels signal the end of a negative energy balance and lipomobilization [57,58]. We found this lipomobilization in late pregnancy to be greater in sheep bearing twins than in those with single pregnancies, as previously reported in New Zeeland meat breeds [6]. Nevertheless, β-OHB and NEFA levels in all our animals remained within physiological levels (0.36–0.80 and 0.18–0.68 mmol/L, respectively) [30,59,60,61], which further corroborates that the nutritional management of our flock was appropriate.

Lamb birth weights were within the physiological range for the Lacaune breed (3.90 ± 0.7 and 4.6 ± 0.2 for female and male lambs, respectively) [62,63]. Males were slightly heavier than females at birth, consistent with previous work [64], but this difference disappeared 17 days later. Metabolic indices for our lambs were always within physiological values and were not affected by lamb sex, except that glucose and triglycerides were higher in males, while cholesterol was higher in females. Given the importance of adequate birth weight on productive performance, we specifically examined phenotypic and metabolic characteristics of lambs with low birth weight (<3 kg). These lambs showed a lower average daily weight gain, as previously reported in meat breeds [65]. At the same time, the male sex could partially overcome the disadvantage of low birth weight: male lambs with a low birth weight increased more in BMI after birth, reaching values similar to those in lambs with normal birth weight, suggesting higher adiposity. The BMI of female lambs with low birth weight did not catch up to their normal-birth-weight counterparts. The metabolic profile was similar between lambs with a normal or low birth weight, except that low-birth-weight lambs showed lower NEFA and urea levels. All these results are consistent with previous work in meat crossbred lambs [66] and may reflect a lower catabolism in low-birth-weight animals because of their smaller body fat and protein reserves [65,67]. 

Lamb birth weight in our study was affected by maternal age and pregnancy rank: lambs were lighter when the mother was a maiden ewe or had a multiple pregnancy. These results are consistent with work in several meat breeds (Finnsheep, Dorset, Rambouillet, Suffolk, and Targhee) [68]. Maternal metabolic indicators showed little effect on lamb phenotype, presumably because maternal indicators were always within physiological ranges. Nevertheless, higher maternal β-OHB levels mid-way through a multiple pregnancy were associated with higher lamb birth weight. This might reflect that sheep bearing twins can better mobilize energy resources to supply more nutrients to the fetus. 

Maternal age affected early postnatal development: lambs born to mature ewes showed a higher average daily weight gain than did lambs born to maiden sheep, confirming results from previous studies in meat ewes [69]. Twin lambs showed a lighter birth weight than single lambs, but they later “caught up” in BW and size. This catching up was less complete in the case of lambs born to maiden sheep with multiple pregnancies, as observed in meat breeds [69]. On the other hand, lamb metabolic indicators were not influenced by maternal age or pregnancy rank, except that lactate and triglycerides were higher in lambs born to maiden sheep with multiple pregnancies than did those lambs born to mature ewes with multiple pregnancies, and urea was higher in lambs from mature ewes. These results suggest greater metabolic stress in lighter lambs, which should be explored in future work. 

In our animals, maternal BCS influenced the lambs primarily in that fatter ewes gave birth to slightly heavier lambs. Heavier ewes gave birth to lambs with slightly more active lipid metabolism (reflected in greater β-OHB concentrations), but other metabolite levels were similar between lambs from heavier or lighter ewes. Studies in Sanjabi ewes have revealed a more extensive influence of maternal BCS on offspring under traditional management conditions [49]. Our observation of milder influence may reflect that the BCS of all ewes in our study were within physiological ranges. 

## 5. Conclusions

In high-yielding Lacaune dairy sheep, appropriate nutritional strategies can maintain maternal BW, BCS, and metabolic profiles within physiological ranges during pregnancy and after lambing, independently of age and pregnancy rank. While pregnancy and lactation place high nutritional and metabolic demands on all ewes, our results suggest that maiden sheep bearing multiple lambs face a greater challenge than maiden sheep with single pregnancies or mature sheep with single or multiple pregnancies. Lamb phenotype and metabolic indices were within physiological ranges almost independently of maternal condition. These results highlight the importance and power of proper nutrition management in dairy flocks. 

## Figures and Tables

**Table 1:** Productive parameters of the farm during the period studied (2016–2018).

Parameters	2016	2017	2018
TMY (l)	319	368	358
YDIM (l/day)	1.62	1.81	1.74
LL (days)	198	202	204
ILI (days)	262	266	269
DPL (days)	63	65	66

TMY: total milk yield per lactation; YDIM: yield per day in milk; LL: lactation length; ILI: interlambing interval; DPL: dry period length.

**Table 2:** Morphometric measurements and metabolic status of lambs, stratified by sex.

Characteristic	Males (*n* = 255)	Females (*n* = 291)
Morphometric Characteristics		
Birth body weight, kg	4.14 ± 0.95 ^a^	3.79 ± 0.86 ^b^
Body weight, 17 days *, kg	8.12 ± 2.24	7.84 ± 2.02
Average daily weight gain, kg	0.238 ± 0.08	0.227 ± 0.06
Birth trunk length, cm	29.41 ± 2.37 ^e^	29.01 ± 2.44 ^f^
Trunk length 17 days *, cm	37.46 ± 4.17	37.49 ± 3.86
Birth BMI-1, kg/m^2^	47.27 ± 6.33 ^a^	44.55 ± 6.89 ^b^
BMI-2, 17 days *, kg/m^2^	57.28 ± 10.84 ^c^	55.20 ± 9.46 ^d^
Thoracic girth, 17 days *, cm	44.38 ± 4.45	44.32 ± 3.95
Abdominal girth, 17 days *, cm	44.55 ± 5.08	43.77 ± 4.66
Metabolic Indices at 17 Days		
Glucose, mg/dL	123.1 ± 26.56 ^e^	120.3 ± 23.54 ^f^
Lactate, mg/dL	19.5 ± 5.40	18.9 ± 5.58
Cholesterol, mg/dL	92.9 ± 18.98 ^e^	96.5 ± 20.06 ^f^
Triglycerides, mg/dL	60.4 ± 36.33 ^e^	54.3 ± 30.41 ^f^
β-OHB, mmol/L	0.134 ± 0.07	0.134 ± 0.06
NEFA, mmol/L	0.533 ± 0.17	0.537 ± 0.16
Urea, mg/dL	31.8 ± 8.09	33.1 ± 9.72

* Lamb age at the second measurement was 17 ± 5 days; BMI: body mass index; β-OHB: β-hydroxybutyrate; NEFA: non-esterified fatty acids. Data are mean ± S.D. Different superscripts within a row denote statistically significant differences (a ≠ b, *p* < 0.0001; c ≠ d, *p* < 0.01; e ≠ f *p* < 0.05).

**Table 3:** Morphometric measurements and metabolic status of lambs, stratified by birth weight and sex.

Parameters	Normal Birth Weight (*n =* 455)	Low Birth Weight (*n* = 122)	Males with Low Birth Weight (*n* = 50)	Females with Low Birth Weight (*n* = 72)
Morphometric Characteristics				
Birth body weight, kg	4.27 ± 0.67 ^a^	2.62 ± 0.42 ^b^	2.66 ± 0.38	2.60 ± 0.46
Body weight, 17 days *, kg	8.39 ± 2.02 ^a^	6.24 ± 1.64 ^b^	6.21 ± 1.49	6.27 ± 1.74
Average daily weight gain, kg	0.237 ± 0.07 ^c^	0.213 ± 0.08 ^d^	0.225 ± 0.10	0.205 ± 0.06
Birth trunk length, cm	29.9 ± 1.73 ^a^	26 ± 2.04 ^b^	25.9 ± 1.81	26.1 ± 2.21
Trunk length 17 days *, cm	38.4 ± 3.49 ^a^	33.6 ± 3.67 ^b^	32.8 ± 3.08	34.2 ± 3.93
Birth BMI-1, kg/m^2^	47.5 ± 5.81 ^a^	38.9 ± 5.77 ^b^	39.6 ± 4.62	38.4 ± 6.43
BMI-2, 17 days *, kg/m^2^	56.5 ± 10.02	54.8 ± 10.72	57.6 ± 11.92 ^c^	53.0 ± 9.53 ^d^
Thoracic girth, 17 days *, cm	45.08 ± 3.93 ^a^	40.83 ± 3.59 ^b^	40.52 ± 3.56	41.03 ± 3.62
Abdominal girth, 17 days *, cm	45.01 ± 4.74 ^a^	40.78 ± 4.21 ^b^	40.91 ± 3.83	40.71 ± 4.47
Metabolic Indices at 17 Days				
Glucose, mg/dL	122.3 ± 25.93	118.4 ± 22.86	121.9 ± 20.00	116.2 ± 23.28
Lactate, mg/dL	19.2 ± 5.52	20.2 ± 5.17	19.6 ± 4.84	20.2 ± 5.40
Cholesterol, mg/dL	92.8 ± 17.99 ^c^	98.1 ± 22.11 ^d^	99.4 ± 21.56	97.3 ± 22.58
Triglycerides, mg/dL	56.6 ± 32.70	63.1 ± 37.78	65.7 ± 43.45	58.5 ± 33.79
β-OHB, mmol/L	0.135 ± 0.07	0.129 ± 0.06	0.134 ± 0.07	0.126 ± 0.06
NEFA, mmol/L	0.545 ± 0.17 ^c^	0.482 ± 0.14 ^d^	0.475 ± 0.14	0.486 ± 0.14
Urea, mg/dL	33.2 ± 8.76 ^e^	31.2 ± 10.27 ^f^	30.2 ± 5.98	31.9 ± 12.23

* Lamb age at the second measurement was 17 ± 5 days. Low birth weight was defined as ≤ 3 kg. BMI: body mass index; β-OHB: β-hydroxybutyrate; NEFA: non-esterified fatty acids. Data are mean ± S.D. Different superscripts within a row denote statistically significant differences (a ≠ b, *p* < 0.0001; c ≠ d, *p* < 0.01; e ≠ f, *p* < 0.05).

**Table 4:** Morphometric measurements and metabolic status of lambs, stratified by maternal age and pregnancy rank.

Parameters	Lambs Born to		Lambs Born as	
	Mature Ewes	Maiden Sheep	Single Pregnancy	Multiple Pregnancy
Morphometric Characteristics	(*n* = 317)	(*n* = 224)	(*n* = 163)	(*n* = 378)
Birth body weight, kg	4.25 ± 0.84 ^a^	3.53 ± 0.87 ^b^	4.28 ± 0.91 ^a^	3.81 ± 0.89 ^b^
Body weight, 17 days *, kg	8.80 ± 2.05 ^a^	6.80 ± 1.63 ^b^	8.06 ± 2.11	7.94 ± 2.14
Average daily weight gain, kg	0.244 ± 0.08 ^a^	0.215 ± 0.07 ^b^	0.229 ± 0.07	0.233 ± 0.07
Birth trunk length, cm	29.93 ± 2.04 ^a^	28.16 ± 2.54 ^b^	29.96 ± 2.14 ^a^	28.87 ± 2.46 ^b^
Trunk length 17 days *, cm	39.17 ± 3.62 ^a^	35.05 ± 3.19 ^b^	37.80 ± 3.91	37.32 ± 4.03
Birth BMI-1, kg/m^2^	47.15 ± 6.62 ^a^	43.85 ± 6.04 ^b^	47.27 ± 7.03 ^a^	45.14 ± 6.55 ^b^
BMI-2, 17 days *, kg/m^2^	56.95 ± 9.65 ^g^	55.20 ± 10.85 ^h^	56.00 ± 10.66	56.33 ± 10.00
Thoracic girth, 17 days *, cm	45.81 ± 3.96 ^a^	42.11 ± 3.61 ^b^	44.43 ± 4.23	44.22 ± 4.23
Abdominal girth, 17 days *, cm	45.87 ± 4.70 ^a^	41.83 ± 4.22 ^b^	44.71 ± 4.68	43.99 ± 5.01
Metabolic Indices at 17 Days				
Glucose, mg/dL	121.6 ± 24.83	121.6 ± 26.16	123.1 ± 29.43	120.9 ± 23.37
Lactate, mg/dL	18.8 ± 5.31 ^e^	20.0 ± 5.53 ^f^	19.2 ± 5.30	19.3 ± 5.49
Cholesterol, mg/dL	94.2 ± 18.04	93.4 ± 20.32	93.5 ± 18.42	94.0 ± 19.27
Triglycerides, mg/dL	54.7 ± 33.20 ^e^	61.6 ± 34.50 ^f^	54.4 ± 27.19	58.9 ± 36.38
β-OHB, mmol/L	0.132 ± 0.06	0.136 ± 0.07	0.131 ± 0.06	0.135 ± 0.07
NEFA, mmol/L	0.542 ± 0.17	0.518 ± 0.15	0.519 ± 0.15	0.538 ± 0.18
Urea, mg/dL	33.9 ± 9.97 ^c^	31.3 ± 7.19 ^d^	32.8 ± 7.70	32.9 ± 9.55

* Lamb age at the second measurement was 17 ± 5 days. BMI: body mass index; β-OHB: β-hydroxybutyrate; NEFA: non-esterified fatty acids. Data are mean ± S.D. Different superscripts within a row denote statistically significant differences (a ≠ b, *p* < 0.0001; c ≠ d, *p* < 0.001; e ≠ f, *p* < 0.005; g ≠ h, *p* < 0.01).

## Supplementary Materials

Figures S1–S8 and Tables S1–S4 are available online at https://www.mdpi.com/2076-2615/9/4/122/s1. **Figure S1**: Body weight (in kg; top panels a, b, and c) and body condition score (BCS; bottom panels d, e, and f) are shown for all ewes (left panels, a and d), mature ewes (central panels, b and e), and maiden sheep (right panels, c and f) throughout the study period. Animals were classified by pregnancy rank (single: ○; multiple: □). Data are mean ± S.E.M. When significant, the time × pregnancy rank interaction is represented in panels by an upper horizontal line and capital letters indicating the significance level (A: *p* < 0.001; B: *p* < 0.05). Asterisks indicate significant differences between single or multiple pregnancies at each time point (**** *p* < 0.0001; *** *p* < 0.001; ** *p* < 0.01; * *p* < 0.05). **Figure S2**: Plasma glucose concentrations (mg/dl) in all ewes (left panels column, a, d, g, and j), mature ewes (central panels column, b, e, h, and k), and maiden sheep (right panels column, c, f, i, and l) throughout the study period. Ewes were classified by pregnancy rank (single: ○; multiple: □; top row of panels a, b, and c) and by level of BCS measured at mid-pregnancy (BCS-1, second row of panels d, e, and f), at late pregnancy (BCS-2; third row of panels g, h, and i), and postpartum (BCS-3; bottom row of panels j, k, and l). BCS levels were “Thin” (▼) for sheep with a BCS ≤ 2, “Average” (♦) for sheep with 2 < BCS < 3, and “Fat” (▲) for sheep with a BCS ≥ 3. Data are mean ± S.E.M. When significant, interactions of time × pregnancy rank or time × BCS level are represented in each panel with an upper horizontal line and capital letters indicating significance level (A: *p* < 0.0001; B: *p* < 0.001; C: *p* < 0.01; D: *p* < 0.05). Asterisks indicate significant differences between groups at each time point (**** *p* < 0.0001; *** *p* < 0.001; ** *p* < 0.01; * *p* < 0.05). **Figure S3**: Plasma lactate concentrations (mg/dl) ) in all ewes (left panels column, a, d, g, and j), mature ewes (central panels column, b, e, h, and k), and maiden sheep (right panels column, c, f, i, and l) throughout the study period. Sheep were classified by pregnancy rank (single: ○; multiple: □; top row of panels a, b, and c) and by level of BCS measured at mid-pregnancy (BCS-1, second row of panels d, e, and f), in late pregnancy (BCS-2; third row of panels g, h, and i), and postpartum (BCS-3; bottom row of panels j, k, and l). BCS levels were “Thin” (▼) for sheep with a BCS ≤ 2, “Average” (♦) for sheep with 2 < BCS < 3, and “Fat” (▲) for sheep with a BCS ≥ 3. Data are mean ± S.E.M. When significant, the interactions of time × pregnancy rank or time × BCS level are represented in each panel with an upper horizontal line and capital letters indicating significance level (A: *p* < 0.0001; B: *p* < 0.001; C: *p* < 0.05; c: tendency). Asterisks indicate significant differences between groups at each time point (** *p* < 0.01; * *p* < 0.05). **Figure S4**: Plasma cholesterol concentrations (mg/dl) in all ewes (left panels column, a, d, g, and j), mature ewes (central panels column, b, e, h, and k), and maiden sheep (right panels column, c, f, i, and l) throughout the study period. Sheep were classified by pregnancy rank (single: ○; multiple: □; top row of panels a, b, and c) and by level of BCS measured at mid-pregnancy (BCS-1, second row of panels d, e, and f), at late pregnancy (BCS-2; third row of panels g, h, and i), and postpartum (BCS-3; bottom row of panels j, k, and l). BCS level was “Thin” (▼) for sheep with a BCS ≤ 2, “Average” (♦) for sheep with 2 < BCS < 3, and “Fat” (▲) for sheep with a BCS ≥ 3. Data are mean ± S.E.M. When significant, the interactions of time × pregnancy rank or time × BCS level are represented in each panel by an upper horizontal line and capital letters indicating significance level (A: *p* < 0.0001; B: *p* < 0.001; C: *p* < 0.05). Asterisks indicate significant differences between groups at each time point (**** *p* < 0.0001; *** *p* < 0.001; ** *p* < 0.01; * *p* < 0.05). **Figure S5**: Plasma triglyceride concentrations (mg/dl) in all ewes (left panels column, a, d, g, and j), mature ewes (central panels column, b, e, h, and k), and maiden sheep (right panels column, c, f, i, and l) throughout the study period. Sheep were classified by pregnancy rank (single: ○; multiple: □; top row of panels a, b, and c) and by level of BCS measured at mid-pregnancy (BCS-1, second row of panels d, e, and f), at late pregnancy (BCS-2; third row of panels g, h, and i), and postpartum (BCS-3; bottom row of panels j, k, and l). BCS levels were “Thin” (▼) for sheep with a BCS ≤ 2, “Average” (♦) for sheep with 2 < BCS < 3, and “Fat” (▲) for sheep with a BCS ≥ 3. Data are mean ± S.E.M. When significant, the interactions of time × pregnancy rank or time × BCS level are represented in each panel with an upper horizontal line and capital letters indicating significance level (A: *p* < 0.001; B: *p* < 0.01). Asterisks indicate significant differences between groups at each time point (**** *p* < 0.0001; *** *p* < 0.001; ** *p* < 0.01; * *p* < 0.05). **Figure S6**: Concentrations of β-hydroxybutyrate (β-OHB) in plasma (mmol/l) in all ewes (left panels column, a, d, g, and j), mature ewes (central panels column, b, e, h, and k), and maiden sheep (right panels column, c, f, i, and l) throughout the study period. Sheep were classified by pregnancy rank (single: ○; multiple: □; top row of panels a, b, and c) and by level of BCS measured at mid-pregnancy (BCS-1, second row of panels d, e, and f), at late pregnancy (BCS-2; third row of panels g, h, and i), and postpartum (BCS-3; bottom row of panels j, k, and l). BCS levels were “Thin” (▼) for sheep with a BCS ≤ 2, “Average” (♦) for sheep with 2 < BCS < 3, and “Fat” (▲) for sheep with a BCS ≥ 3. Data are mean ± S.E.M. When significant, the interactions of time × pregnancy rank or time × BCS level are represented in each panel with an upper horizontal line and capital letters indicating significance level (A: *p* < 0.0001; B: *p* < 0.001). Asterisks indicate significant differences between groups at each time point (**** *p* < 0.0001; *** *p* < 0.001; ** *p* < 0.01; * *p* < 0.05). **Figure S7**: Concentrations of non-esterified fatty acids (NEFA) in plasma (mmol/l) in all ewes (left panels column, a, d, g, and j), mature ewes (central panels column, b, e, h, and k), and maiden sheep (right panels column, c, f, i, and l) throughout the study period. Sheep were classified by pregnancy rank (single: ○; multiple: □; top row of panels a, b, and c) and by level of BCS measured at mid-pregnancy (BCS-1, second row of panels d, e, and f), at late pregnancy (BCS-2; third row of panels g, h, and i), and postpartum (BCS-3; bottom row of panels j, k, and l). BCS levels were “Thin” (▼) for sheep with a BCS ≤ 2, “Average” (♦) for sheep with 2 < BCS < 3, and “Fat” (▲) for sheep with a BCS ≥ 3. Data are mean ± S.E.M. When significant, the interactions of time × pregnancy rank or time × BCS level are represented in each panel by an upper horizontal line and capital letters indicating significance level (A: *p* < 0.0001; B: *p* < 0.001; b: tendency). Asterisks indicate significant differences between groups at each time point (**** *p* < 0.0001; *** *p* < 0.001; ** *p* < 0.01; * *p* < 0.05). **Figure S8**: Plasma urea concentration (mg/dl) in all ewes (left panels column, a, d, g, and j), mature ewes (central panels column, b, e, h, and k), and maiden sheep (right panels column, c, f, i, and l) throughout the study period. Sheep were classified by pregnancy rank (single: ○; multiple: □; top row of panels a, b, and c) and by level of BCS measured at mid-pregnancy (BCS-1, second row of panels d, e, and f), at late pregnancy (BCS-2; third row of panels g, h, and i), and postpartum (BCS-3; bottom row of panels j, k, and l). BCS levels were “Thin” (▼) for sheep with a BCS ≤ 2, “Average” (♦) for sheep with 2 < BCS < 3, and “Fat” (▲) for sheep with a BCS ≥ 3. When significant, the interactions of time × pregnancy rank or time × BCS level are represented in each panel with an upper horizontal line and capital letters indicating significance level (A: *p* < 0.0001; B: *p* < 0.001; C: *p* < 0.05; c: tendency). Asterisks indicate significant differences between groups at each time point (**** *p* < 0.0001; *** *p* < 0.001; ** *p* < 0.01; * *p* < 0.05). **Table S1**: Morphometric measurements and metabolic status of lambs, stratified by maternal age and pregnancy rank. **Table S2**: Morphometric measurements and metabolic status of lambs, stratified by maternal BCS in mid- or late pregnancy. **Table S3:** Pearson correlation coefficients between lamb birth weight and maternal metabolic indices at mid-gestation, with mothers classified by maternal age, pregnancy rank, maternal BCS at mid-pregnancy, and BCS in late pregnancy. **Table S4:** Pearson correlation coefficients between lamb birth weight and maternal metabolic indices in late gestation, with mothers classified by maternal age, pregnancy rank, maternal BCS at mid-pregnancy, and BCS in late pregnancy.
